# Epigenetic therapy of myelodysplastic syndromes connects to cellular differentiation independently of endogenous retroelement derepression

**DOI:** 10.1186/s13073-019-0707-x

**Published:** 2019-12-23

**Authors:** Anastasiya Kazachenka, George R. Young, Jan Attig, Chrysoula Kordella, Eleftheria Lamprianidou, Emmanuela Zoulia, George Vrachiolias, Menelaos Papoutselis, Elsa Bernard, Elli Papaemmanuil, Ioannis Kotsianidis, George Kassiotis

**Affiliations:** 1 Retroviral Immunology, The Francis Crick Institute, 1 Midland Road, London, NW1 1AT UK; 2 Retrovirus-Host Interactions, The Francis Crick Institute, The Francis Crick Institute, 1 Midland Road, London, NW1 1AT UK; 3 Department of Haematology, Democritus University of Thrace Medical School, Alexandroupolis, Greece; 4 Center for Molecular Oncology, Center for Heme Malignancies and Department of Epidemiology and Biostatistics, Memorial Sloan Kettering Cancer Center, New York, NY 10065 USA; 5 Department of Medicine, Faculty of Medicine, Imperial College London, London, W2 1PG UK

## Abstract

**Background:**

Myelodysplastic syndromes (MDS) and acute myeloid leukaemia (AML) are characterised by abnormal epigenetic repression and differentiation of bone marrow haematopoietic stem cells (HSCs). Drugs that reverse epigenetic repression, such as 5-azacytidine (5-AZA), induce haematological improvement in half of treated patients. Although the mechanisms underlying therapy success are not yet clear, induction of endogenous retroelements (EREs) has been hypothesised.

**Methods:**

Using RNA sequencing (RNA-seq), we compared the transcription of EREs in bone marrow HSCs from a new cohort of MDS and chronic myelomonocytic leukaemia (CMML) patients before and after 5-AZA treatment with HSCs from healthy donors and AML patients. We further examined ERE transcription using the most comprehensive annotation of ERE-overlapping transcripts expressed in HSCs, generated here by de novo transcript assembly and supported by full-length RNA-seq.

**Results:**

Consistent with prior reports, we found that treatment with 5-AZA increased the representation of ERE-derived RNA-seq reads in the transcriptome. However, such increases were comparable between treatment responses and failures. The extended view of HSC transcriptional diversity offered by de novo transcript assembly argued against 5-AZA-responsive EREs as determinants of the outcome of therapy. Instead, it uncovered pre-treatment expression and alternative splicing of developmentally regulated gene transcripts as predictors of the response of MDS and CMML patients to 5-AZA treatment.

**Conclusions:**

Our study identifies the developmentally regulated transcriptional signatures of protein-coding and non-coding genes, rather than EREs, as correlates of a favourable response of MDS and CMML patients to 5-AZA treatment and offers novel candidates for further evaluation.

## Background

Myelodysplastic syndromes (MDS) and acute myeloid leukaemia (AML) are characterised by abnormal differentiation of bone marrow haematopoietic stem cells (HSCs) into immature CD34^+^ blast cells and ineffective haematopoiesis [1]. Genetic abnormalities are frequently observed in these bone marrow HSC cancers, including deletion of chromosome 5q, and mutations in genes implicated in RNA splicing, cell signalling, DNA modification and chromatin regulation [2–5].

Consistent with driver mutations affecting epigenetic modifications, aberrant DNA methylation patterns, particularly DNA hypermethylation in promoters of tumour suppressor genes, are considered central to the pathogenesis of MDS and progression to AML [6–8]. Accordingly, epigenetic drugs (epidrugs) that can reverse the repressive state of DNA hypermethylation, such as 5-azacytidine (5-AZA) and 5-aza-2′-deoxycytidine (decitabine), have been the mainstay of treatment for higher-risk MDS and also for older, unfit AML patients. Being cytidine analogues, both 5-AZA and decitabine are incorporated into the DNA of highly proliferating cells leading to a genome-wide decrease of methylation levels, whereas 5-AZA is additionally incorporated into RNA [9, 10]. However, the mechanisms by which inhibition of DNA methylation or additional effects of epidrug therapy might ultimately lead to clinical remission and restoration of normal haematopoiesis in MDS or chronic myelomonocytic leukaemia (CMML) patients remain incompletely understood. Indeed, a favourable outcome of 5-AZA treatment is observed in less than half of treated patients, almost all of whom also relapse [11, 12], and these disparate responses cannot yet be predicted.

Several models of epidrug therapeutic modes of action have been proposed [9, 13–19], some of which incriminate endogenous retroelements (EREs), which occupy a substantial fraction of the genome [20, 21]. EREs are divided into long-terminal repeat (LTR) elements, which include human endogenous retroviruses (HERVs) and mammalian apparent LTR-retrotransposons (MaLRs), and non-LTR elements, which include long and short interspersed nuclear elements (LINEs and SINEs, respectively) and composite SINE-VNTR-Alu (SVA) elements [22, 23]. There are over four million ERE integrations in the human genome, but the damaging effects arising from their transcriptional utilisation are minimised by dedicated epigenetic and splicing repression mechanisms [22].

Studies with 5-AZA treatment of human cancer cell lines in vitro or a murine ovarian cancer model in vivo indicate that epigenetic derepression of EREs triggers innate immune pathways, through the production of double-stranded RNA (dsRNA), thereby mimicking viral infection [24–27]. Moreover, 5-AZA treatment of primary MDS HSCs in vitro has been correlated with the upregulation of certain HERVs [28], and loss of the histone methyltransferase SETDB1 derepressed EREs and triggered innate immunity through the production of dsRNA in human AML cell lines in vitro [29]. These observations suggest a model whereby ‘viral mimicry’ of EREs transcriptionally induced by 5-AZA treatment triggers an antiviral response state, typified by interferon (IFN) I production, which in turn determines the therapeutic outcome. However, direct evidence to support this hypothesis or indeed a correlation between ERE modulation and treatment outcome from in vivo epidrug treatment of MDS or CMML patients is currently lacking.

We set out to test this hypothesis by determining the pattern of ERE expression in response to 5-AZA therapy in vivo in bone marrow HSCs isolated from MDS and CMML patients. Using optimised bioinformatics pipelines and de novo transcript assembly, we captured the most complete view of ERE expression and transcriptional diversity in healthy and dysplastic HSCs to date. Our results do not support a role for ERE modulation in the therapeutic response to 5-AZA. Instead, they suggest that HSC differentiation states, reflected in the diversity of captured alternatively spliced variants of developmentally regulated genes, are predictive of 5-AZA treatment outcome and provide candidates for further evaluation.

## Methods

### Patients and sample collection

This study includes 2 cohorts of samples. The first cohort consists of BM aspirates from 4 healthy individuals and 12 patients diagnosed with AML, MDS or CMML (Additional file 1: Table S1 and Table S2). The second cohort includes 5 healthy individuals and 17 patients diagnosed with AML, MDS or CMML (Additional file 1: Table S1 and Table S2). Median patient ages, at treatment initiation, were 72 and 70 for MDS and CMML, respectively, and 62 and 60 for healthy volunteers and AML patients, respectively. MDS and CMML patients from both cohorts were treated with 5-AZA for 6 cycles. Samples from the first cohort were obtained before and, on average, 15 days after the start of 1 round of treatment and were used for the isolation of CD34^+^ HSCs. Samples from the second cohort were obtained before and, on average, 15 days after the start of 1 and 6 rounds of treatment and were used for the isolation of CD34^+^ HSCs, CD4^+^ T cells and CD8^+^ T cells. Patient responses were assessed according to the International Working Group 2006 criteria [30] and were selected for either a complete response (CR) or failure to respond (FAIL), to allow clear patient stratification based on treatment outcome. As complete remission following 5-AZA treatment is rarely observed, our criteria for CR included patients exhibiting complete remission with incomplete haematologic recovery (neutrophil counts marginally lower than 10^9^/L). Also, all treatment failures exhibited progressive disease despite treatment, thus representing true refractoriness to 5-AZA therapy, as opposed to failure due to intractable toxicity or death. All patients were recruited at the General University Hospital of Alexandroupolis, Greece, and samples were obtained with written informed consent and approval of the relevant institutional human research ethics committees.

### Cell sorting

Bone marrow aspirates from healthy individuals and patients diagnosed with AML, CMML or MDS before and after 1 or 6 cycles of 5-AZA treatment were stained for 20 min at room temperature or at 4 °C with the following directly conjugated antibodies: CD8 PECy7 (anti-human CD8, clone 3B5, Cat# MHCD0812, Thermo Fisher Scientific), CD4 Pacific Blue (anti-human CD4 Antibody clone OKT4, Cat# 317402, Thermo Fisher Scientific), CD34 PE (anti-human CD34, clone 4H11 Cat# 12-0349-42, eBioscience) and CD45 FITC (anti-human CD45 FITC clone HI30 Cat# 11-0459-42, eBioscience). CD34^+^ HSCs, CD4^+^ T cells and CD8^+^ T cells were identified using the gating strategy depicted in Additional file 2: Figure S1. Cell populations were purified (> 98% purity) by cell sorting performed on a FACSAria Fusion flow cytometer (BD Biosciences) or MoFlo cell sorters (Dako-Cytomation).

### Transcriptional profiling by RNA-seq

The SMART-Seq v4 Ultra Low Input RNA Kit (Takara, Kusatsu, Japan) was used for cDNA synthesis from intact cells according to the manufacturer’s protocol and libraries sequences using Illumina HiSeq machines (PE150). Data were deposited at the EMBL-EBI repository (www.ebi.ac.uk/arrayexpress) under accession number E-MTAB-8208. Quality of raw sequencing data was assessed by FastQC v0.11.5. Adapter and quality trimming (Q20) was conducted using BBDuk2 (BBMap v36.20) from BBTools (http://jgi.doe.gov/data-and-tools/bb-tools/) followed by Trimmomatic v0.36 [31]. The resulting paired-end reads were aligned to GRCh38/hg38 using HISAT2 v2.1.0 [32]. FeatureCounts (part of the Subread package v1.5.0 [33]) was used to the calculate gene and repeat expression (including only uniquely mapping reads) using GENCODE.v24 basic [34] and EREs annotated by RepeatMasker v4.06 configured with HMMER 3.1b2 and using Dfam2 HMM libraries. DESeq2 v1.22.1 within R v3.5.1 [35] was used for the read count normalisation for sequencing depth across samples. All downstream differential expression analysis and visualisation were carried out using Qlucore Omics Explorer 3.3 (Qlucore, Lund, Sweden).

### Additional datasets

In addition to the datasets generated here, we have analysed RNA-seq data from human CD34^+^ HSCs [16] previously deposited at SRA (www.ncbi.nlm.nih.gov/sra) under accession number SRP067631. We have also analysed microarray data from normal human haematopoiesis [36], obtained from the BloodSpot data portal (www.bloodspot.eu), with the original data available at the GEO repository (www.ncbi.nlm.nih.gov/geo) under accession number GSE42519.

### HSC de novo transcriptome assembly

Sixty-four RNA-seq datasets generated for CD34^+^ HSCs purified from bone marrow aspirates of healthy individuals and patients diagnosed with MDS, CMML or AML were used to de novo assemble the transcriptome. RNA-seq reads were adapter trimmed and length filtered (both reads of the pair ≥ 35 nucleotides) using Cutadapt v1.9.1 [37]. Digital normalisation (*k* = 20, max depth = 200, min depth = 3) using khmer v1.4.1 [38] was performed for RNA-seq datasets split by an individual condition into 4 groups (healthy, MDS, CMML and AML). Reads were aligned to GRCh38/hg38 using HISAT2 v2.1.0 [32] and genome-guided assembly conducted using Trinity v2.2.0 [39] with in silico depth normalisation disabled. Contigs in resulting assemblies were polyA-trimmed and entropy-filtered (≥ 0.7) using trimpoly (SeqClean v110222, https://sourceforge.net/projects/seqclean/) and BBDuk2 (http://jgi.doe.gov/data-and-tools/bb-tools/), respectively. The original RNA-seq datasets were quasi-mapped to the corresponding assembly using Salmon v0.11.4 [40]. Only contigs that were expressed ≥ 0.05 TPM in at least 1 sample were left for further mapping to GRCh38/hg38 using GMAP v2016-11-07 [41], where contigs aligning with ≤ 85% identity over ≤ 85% of their length were removed. The resulting 4 assemblies were flattened and merged together using gffread (Cufflinks v2.2.1) [42]. Transcript expression was quantified using Salmon v0.11.4 [40] and differential expression analysis and visualisation carried out using Qlucore Omics Explorer 3.3 (Qlucore, Lund, Sweden). Cuffcompare (Cufflinks 2.2.1) [43] and custom R scripts were used to annotate the transcripts against GENCODE v29 (comprehensive gene annotation) [34] and to compare to ISO-seq transcripts.

### Full-length mRNA sequencing of dysplastic HSCs

Two samples were prepared for full-length mRNA sequencing (ISO-seq). The first sample was a pool of CD34^+^ HSCs cells from five MDS patients before 5-AZA treatment (GEO531A16, GEO531A13, GEO531A5, GEO531A11 and GEO531A3), and the second a pool of two untreated AML and two untreated CMML patients (GEO531A2, GEO531A9, GEO531A6 and GEO531A7). Total RNA was extracted using the Qiagen RNeasy Mini Kit. RNA yields and RIN scores were assessed on the Agilent Bioanalyzer (Agilent, Santa Clara, USA). Both samples were sequenced on a single Pacific Biosciences (Menlo Park, USA) Sequel SMRT cell by GeneWiz (South Plainfield, USA). Data were deposited at the EMBL-EBI repository (www.ebi.ac.uk/arrayexpress) under accession number E-MTAB-8195. PacBio tools were used for downstream analysis and de novo isoform discovery (https://github.com/PacificBiosciences/pbbioconda), and identified isoforms were aligned to GRCh38/hg38 using GMAP v2016-11-07 [41]. The resulting GFF3 files were merged together using gffread (Cufflinks v2.2.1) [42]. Cuffcompare (Cufflinks 2.2.1) [43] was used to compare the identified transcripts to GENCODE v29 (comprehensive gene annotation) [34] and the de novo transcriptome assembly.

### Expression analyses by quantitative real-time reverse transcription-based PCR

The level of transcription of the selected de novo assembled isoforms was quantified by quantitative real-time reverse transcription-based PCR (qRT-PCR). RNA was purified from sorted bone marrow HSC lysates using the RNAeasy mini QIAcube Kit (Qiagen). DNA digestion was performed using RNase-Free DNase Set (Qiagen) and cDNA prepared using the High-Capacity cDNA Reverse Transcription Kit (Life Technologies). PCR primers were designed using Primer3 software and are shown in Additional file 1: Table S3 and qRT-PCR performed using Fast SYBR Green Master Mix (Thermo Fisher Scientific) on QuantStudio instruments. Relative cDNA abundance was calculated using the ΔCT method and normalised to HPRT expression.

### Gene functional annotation

Pathway analyses were performed using the Database for Annotation, Visualization and Integrated Discovery (DAVID) v6.8 (https://david.ncifcrf.gov/home.jsp).

### Survival analysis

Correlation of AML survival probability was calculated using the BloodSpot data portal (www.bloodspot.eu) with expression data from microarray analysis of AML in a TCGA cohort of 172 AML patients [44].

### Statistical analyses

Statistical comparisons were made using SigmaPlot 14 (Systat Software Inc.). Parametric comparisons of normally distributed values that satisfied the variance criteria were made by unpaired Student’s *t* tests or one-way ANOVAs. Data that did not pass the variance test were compared with non-parametric two-tailed Mann-Whitney rank sum test or ANOVA on rank tests. Analysis of processed RNA-seq data, hierarchical clustering and heat-map production was with Qlucore Omics Explorer 3.3 (Qlucore, Lund, Sweden).

## Results

### Gene and ERE transcription differentiates dysplastic HSCs

**Fig. 1:**
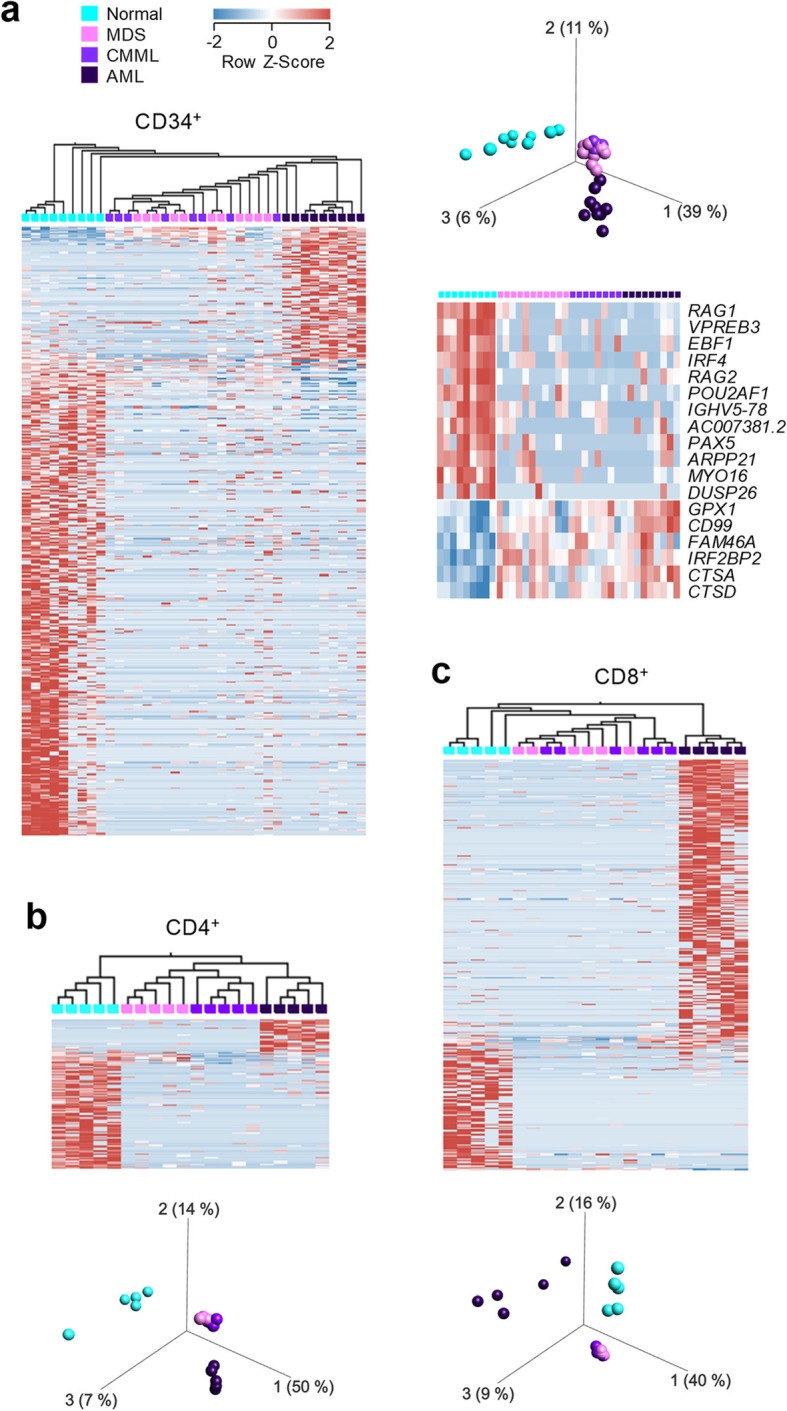
Differential gene and ERE expression in healthy and dysplastic bone marrow cells. **a** Gene and ERE transcripts that are differentially expressed between healthy and untreated dysplastic HSCs were identified by multigroup comparison (*q* ≤ 0.05). Heatmap of the expression of all the identified genes and EREs and hierarchical clustering of samples according to this expression (left), principal component analysis (PCA) based on this expression (top right) and heatmap of the expression of selected genes (bottom right). HSCs from all untreated patients from both cohorts are shown. **b**, **c** Heatmaps and corresponding PCA plots of gene and ERE expression differentiating CD4^+^ T cells (**b**) or CD8^+^ T cells (**c**) isolated from healthy and untreated dysplastic bone marrow aspirates (multigroup comparison, *q* ≤ 0.05). Only patients from the second cohort are shown, as RNA-seq data from bone marrow T cells from the first cohort are not available

In order to discern the transcriptional profiles of healthy and dysplastic HSCs, as well as their response to 5-AZA therapy, we compared HSCs from MDS and chronic myelomonocytic leukaemia II (referred to here as CMML) patients, with known mutational signatures, prior to and at defined time points after 5-AZA treatment (Additional file 1: Table S1 and Table S2). For comparison, we also included healthy volunteers and untreated de novo AML (referred to here as AML) patients (Additional file 1: Table S1 and Table S2). RNA-seq data generated from highly purified bone marrow CD34^+^ HSCs (Additional file 2: Figure S1) were analysed using a previously established pipeline that quantifies the transcription of repetitive elements together with annotated genes [45]. This analysis distinguished healthy and dysplastic HSCs by the transcription of 479 elements (Additional file 1: Table S4), which included 75 genes (*q* ≤ 0.05) (Fig. 1a). Genes upregulated in healthy HSCs included several involved in B cell differentiation, such as *RAG1* and *RAG2* mediating immunoglobulin gene segment recombination, and the B cell-specific transcription factor *PAX5*, whereas those upregulated in AML, and to a lesser extent in MDS and CMML HSCs were involved in myeloid differentiation, such as the cathepsins *CTSA* and *CTSD* (Fig. 1a). MDS and CMML HSCs were transcriptionally indistinguishable, although, overall, they were distinct from both healthy and AML HSCs (Fig. 1a). Transcriptional differences between healthy, MDS, CMML and AML cells permeated also in CD4^+^ and CD8^+^ T cells purified from the same bone marrow biopsies, with MDS and CMML being the closest (Fig. 1b, c). Differentially expressed elements included mostly repetitive elements with only 3 of 107 and 25 of 346 elements corresponding to annotated genes in CD4^+^ and CD8^+^ T cells, respectively (*q* ≤ 0.05). These data highlighted the transcriptional commonalities between MDS and CMML, which were, therefore, combined in subsequent analyses.

### Therapeutic response to 5-AZA independently of ERE transcriptional induction

**Fig. 2:**
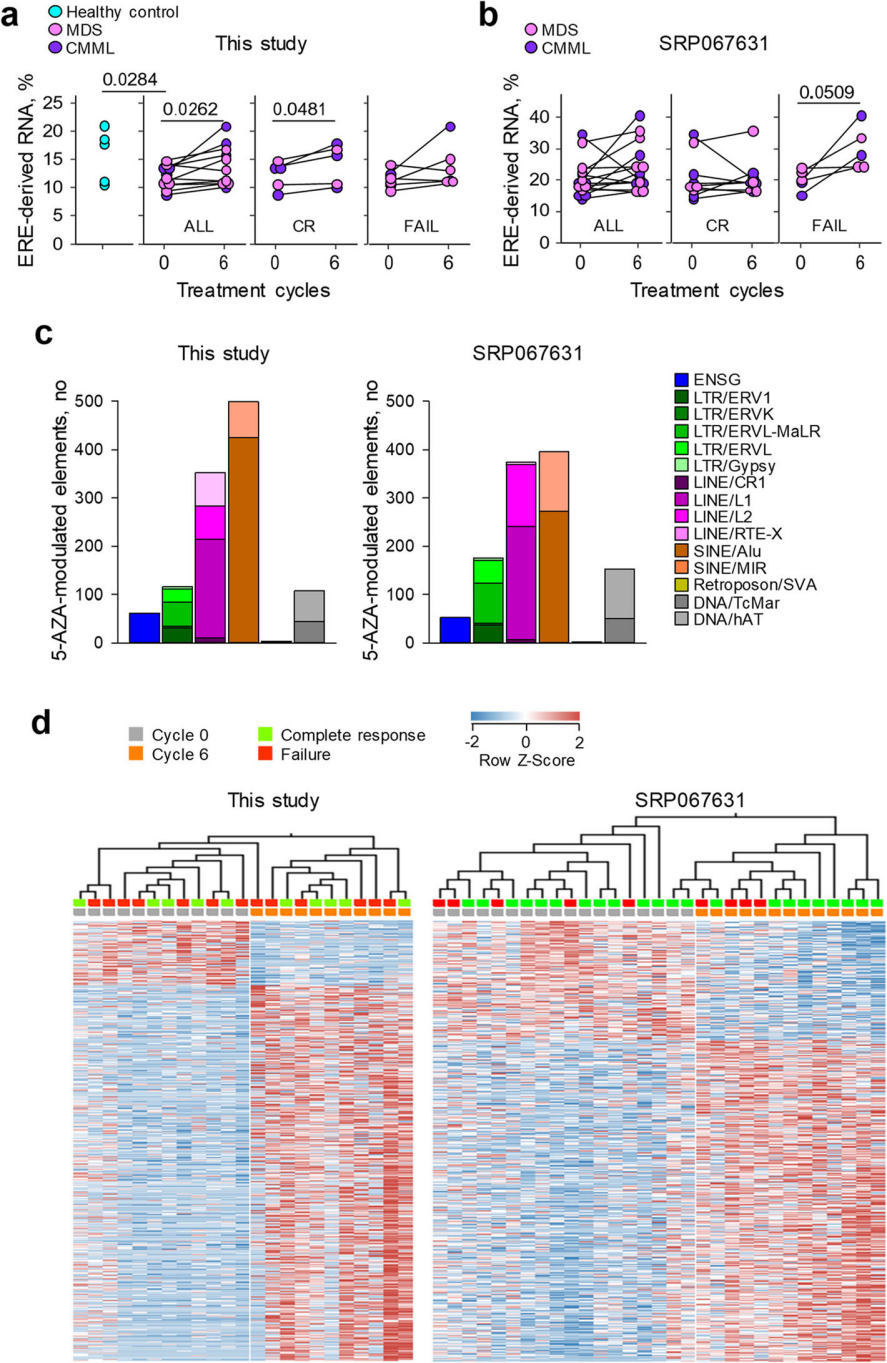
ERE responsiveness to 5-AZA treatment. **a** Comparison of total ERE-derived RNA-seq reads before and after 6 cycles of 5-AZA treatment in MDS and CMML bone marrow HSCs, compared with healthy HSCs. Only patients from the second cohort are shown, as the first cohort was not sampled after six treatment cycles, and lines connect the values for a given patient. Patients with a complete response (CR) or treatment failure (FAIL) are also plotted separately. **b** Comparison of total ERE-derived RNA-seq reads before and after 6 cycles of 5-AZA treatment in a previously published MDS and CMML bone marrow HSC dataset (SRP067631). **c** Representation of different classes among 5-AZA-responsive elements in our study (left) and in SRP067631 (right) (≥ 2-fold, *p* ≤ 0.006). **d** Hierarchically clustered heatmap of 5-AZA-responsive elements in MDS and CMML bone marrow HSCs in our study (left) and in SRP067631 (right) (≥ 2-fold, *p* ≤ 0.006). Only patients from the second cohort of our study are shown

To examine the possible effect of 5-AZA on ERE transcription in vivo, we first calculated the combined proportion of ERE-derived RNA-seq reads. In healthy donor HSCs, ERE-derived reads added up to ~ 16% of all sequenced reads (Fig. 2a), suggesting that ERE-containing transcripts formed a sizeable proportion of the total transcriptome. Consistent with their transcriptionally repressed state [6, 8, 46], transcriptomes from untreated MDS and CMML HSCs exhibited a reduced proportion of ERE-derived reads, compared with those from healthy control HSCs (Fig. 2a). ERE transcription in MDS and CMML HSCs was significantly increased by the sixth cycle of 5-AZA treatment, to levels equivalent to those in healthy donor HSCs (Fig. 2a), indicating at least partial restoration of gene and ERE expression patterns following treatment. However, the representation of EREs in the transcriptome was increased by 5-AZA treatment in patients with treatment failure and with a complete response, albeit only the latter reached a statistical significance cutoff of ≤ 0.05 (Fig. 2a).

To extend these initial findings, we analysed an independently generated dataset (accession number: SRP067631), which also included MDS and CMML patient samples prior to and at the sixth cycle of 5-AZA treatment, although a corresponding group of healthy donor samples was not available in this study [16]. The overall representation of EREs in the transcriptomes calculated by our pipeline was higher in the SRP067631 than in our cohort (Fig. 2a, b), likely due to the methodological differences in RNA-seq. Nevertheless, ERE representation was similar between patients at the sixth cycle of 5-AZA treatment in the SRP067631 cohort, irrespective of treatment outcome, and measurable increases in ERE representation were observed also in patients with treatment failure (Fig. 2b).

These results indicated that the therapeutic response to 5-AZA did not correlate with the global expression of EREs, but they did not preclude the possibility that treatment outcome correlated with the modulation of specific ERE groups or loci. To test this hypothesis, we first examined the composition of elements that were most responsive to 5-AZA treatment in our cohort and in SRP067631 (1095 and 1164, respectively, at *p* ≤ 0.006) (Fig. 2c). These included annotated genes and highly diverse groups of LTR and non-LTR elements (Fig. 2c), reflecting genomic diversity [20]. However, 5-AZA responsive elements were similarly expressed prior to treatment and were similarly modulated following treatment between patients irrespective of 5-AZA treatment outcome, as no clustering of patients according to treatment outcome was observed (Fig. 2d).

We further examined the expression of individual LTR elements that were previously found induced by 5-AZA in cell lines [24, 25, 28, 47, 48]; the groups of LTR elements, to which these individual loci belonged; and groups of LTR elements associated with overall survival in AML [49] (Additional file 2: Figure S2 and Figure S3). None of the individual loci examined was upregulated or otherwise modulated by 5-AZA treatment, with the possible exception of *ERVFRD-1* (encoding Syncytin 2), expression of which was downregulated following 5-AZA treatment, particularly when treatment failed (Additional file 2: Figure S2). At the group level, four groups of LTR elements (*MER54A*, *MLT1B*, *LTR12* and *LTR24C*) were significantly overrepresented following 5-AZA treatment only in responders, whereas five groups (*MER21C*, *ERV-16A3_LTR*, *MLT1A0*, *MLT1C2* and *THE1D*) were overrepresented following 5-AZA treatment irrespective of the outcome (Additional file 2: Figure S3).

Although EREs appeared induced by 5-AZA regardless of the treatment outcome, it remained possible that they triggered an antiviral response only in patients with a complete response to treatment. To investigate this possibility, we assessed the transcription of genes and LTR elements known to be responsive to IFN. These included a list of 108 LTR elements (Additional file 1: Table S5) that were previously shown to be induced in haematopoietic cells from systemic lupus erythematosus patients and by IFN-β treatment of multiple sclerosis patients [45]. Analysis of these IFN-inducible LTR elements failed to reveal transcriptional induction after 6 cycles of 5-AZA treatment in our cohort or in SRP067631, or a correlation with treatment outcome (Additional file 2: Figure S4a). We have also analysed the expression of a compilation of 58 typical IFN signature genes (ISGs) (Additional file 1: Table S5), which also failed to reveal responsiveness to 5-AZA therapy or correlation with its outcome (Additional file 2: Figure S4b). Next, we have analysed a list of 401 genes, which were previously shown to be induced by 5-AZA in vitro treatment in one or more cell lines [50], which included ISGs; cytokine and chemokine genes; genes involved in antigen presentation, inflammation or antiviral defence; and cancer-testis antigen genes (collectively referred to here as 5-AZA ISGs). Analysis of the latter set of genes corroborated the lack of responsiveness to 6 cycles of 5-AZA therapy or correlation with its outcome in our cohort or in SRP067631 (Additional file 2: Figure S4c).

As an IFN response to innate immune stimulation could be transient and also subject to negative-feedback regulation, we next examined whether sampling after 6 cycles of 5-AZA treatment might have missed earlier waves of ISG induction. However, using HSCs samples acquired as early as 6 days after the end of the first cycle of 5-AZA treatment did not provide any evidence for elevated transcription of IFN-inducible LTR elements, typical ISGs or 5-AZA ISGs (Additional file 2: Figure S4d-f).

In their independent analysis, Unnikrishnan et al. also failed to detect upregulation of ISGs in patients who responded to 6 cycles of 5-AZA treatment [16]. They, nevertheless, identified a list of 302 genes induced by 5-AZA preferentially in responders, and these included inflammation-related genes [16]. Our reanalysis of the SRP067631 cohort confirmed significantly higher induction of these 302 genes (using their average expression as an index) only in samples from responders, in agreement with the original analysis [16]. However, a similar analysis of our new cohort did not offer evidence for significant upregulation in responders from the current study (Additional file 2: Figure S5). Collectively, these findings argued against sustained innate immune activation specifically in CD34^+^ HSCs as a correlate or predictor of the therapeutic response to 5-AZA treatment.

Given its unexpected nature, we sought possible explanations for the apparent lack of an IFN or inflammatory response in CD34^+^ HSCs following 5-AZA treatment. A general property of diverse types of stem cell is the constitutive expression of certain ISGs and their refractoriness to IFN stimulation [51]. This property is lost during cellular differentiation, when a constitutive expression of ISGs is reduced and cells become responsive to IFN stimulation [51]. It was, therefore, conceivable that the apparent lack of ERE induction and IFN response following 5-AZA treatment was due to this property of CD34^+^ HSCs. Indeed, compared with CD4^+^ and CD8^+^ T cells purified from the same bone marrow aspirates, CD34^+^ HSCs exhibited significantly elevated expression of 5-AZA ISGs and of 5-AZA-responsive ERVs in healthy donors and in untreated MDS, CMML and AML patients in our cohort (Additional file 2: Figure S6). Thus, the increased expression of ERVs and ISGs in healthy and dysplastic CD34^+^ HSCs prior to treatment may have blunted additional induction after treatment.

### Assessing the complexity of CD34^+^ HSC transcriptome by de novo assembly

Our analysis of RNA-seq reads mapping to EREs did indicate increased representation of *MER54A*, *MLT1B*, *LTR12* and *LTR24C* EREs, consistent with prior reports [24, 25, 28, 47–49]. However, this type of analysis captures the aggregate transcription of all ERE integrations belonging to each of these groups, regardless of the transcript to which they belong. In many cases, EREs are part of gene transcripts, as terminal exons or embedded in 3′ untranslated regions (UTRs) [52]. Therefore, the increased representation of ERE reads may not be due to the genuine upregulation of ERE transcription per se, but rather an upregulation of the gene transcript, in which the ERE is embedded.

**Fig. 3:**
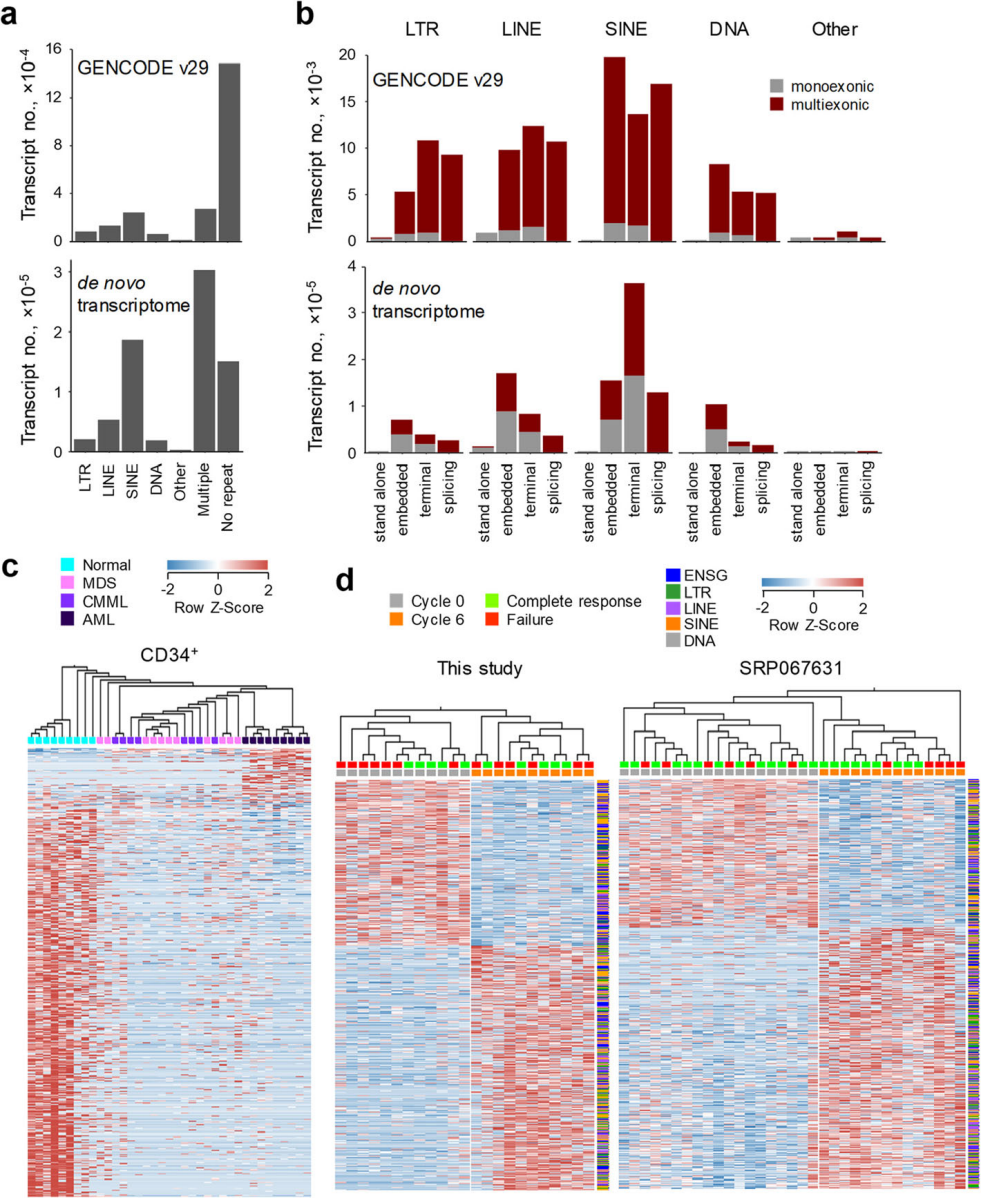
De novo transcript assembly of healthy and dysplastic HSCs. **a** Number of transcripts overlapping repeats in GENCODE v29 (comprehensive gene annotation) and the de novo assembled transcriptome of healthy, MDS, CMML and AML HSCs. **b** Representation of multiexonic or monoexonic repeat-overlapping transcripts, according to the transcript structure in GENCODE v29 and the de novo assembled transcriptome. **c** De novo assembled transcripts that are differentially expressed between healthy and untreated dysplastic HSCs were identified by multigroup comparison (*q* ≤ 0.05). Heatmap of the expression of all the identified transcripts and hierarchical clustering of samples according to this expression. HSCs from all untreated patients from both cohorts are shown. **d** Hierarchically clustered heatmap of 5-AZA-responsive de novo-assembled transcripts in MDS and CMML bone marrow HSCs in our study (left) and in SRP067631 (right) (≥ 2-fold, *p* ≤ 0.006). Only patients from the second cohort of our study are shown

As accurate quantitation of ERE transcription requires knowledge of the transcripts including EREs, many of which might not be annotated, we de novo assembled the transcriptomes of healthy and dysplastic CD34^+^ HSCs. This process generated a total of 730,242 expressed transcripts, of which the majority (420,594) were multiexonic; 26,691 were previously fully annotated, and 703,551 were partially annotated or unannotated, when compared to GENCODE [34]. The increased number of de novo assembled transcripts was mainly due to transcripts overlapping SINEs or multiple EREs, with modest increases in other types of transcripts (Fig. 3a). Within ERE-overlapping transcripts, those consisting of stand-alone EREs were the smallest minority, with a majority being chimeric transcripts with EREs either embedded or acting as a terminal exon (Fig. 3b).

Assessment of expression of de novo assembled transcripts identified 868 elements that distinguish healthy and dysplastic HSCs (*q* ≤ 0.05), again with MDS and CMML characterised by comparably reduced expression (Fig. 3c). However, despite extending the transcriptome in general and the representation of EREs in particular, the transcripts that were most responsive to 5-AZA treatment in our cohort and in SRP067631 (1393 and 2081, respectively, at *p* ≤ 0.006) did not distinguish patients with a clinical response from those with failure (Fig. 3d). This was consistent with our previous analysis of repetitive element expression (Fig. 2d) and further argued against the induction of ERE transcription as a cause or correlate of the therapeutic response to 5-AZA.

### ISO-seq highlights alternatively spliced isoforms in CD34^+^ HSCs

**Fig. 4:**
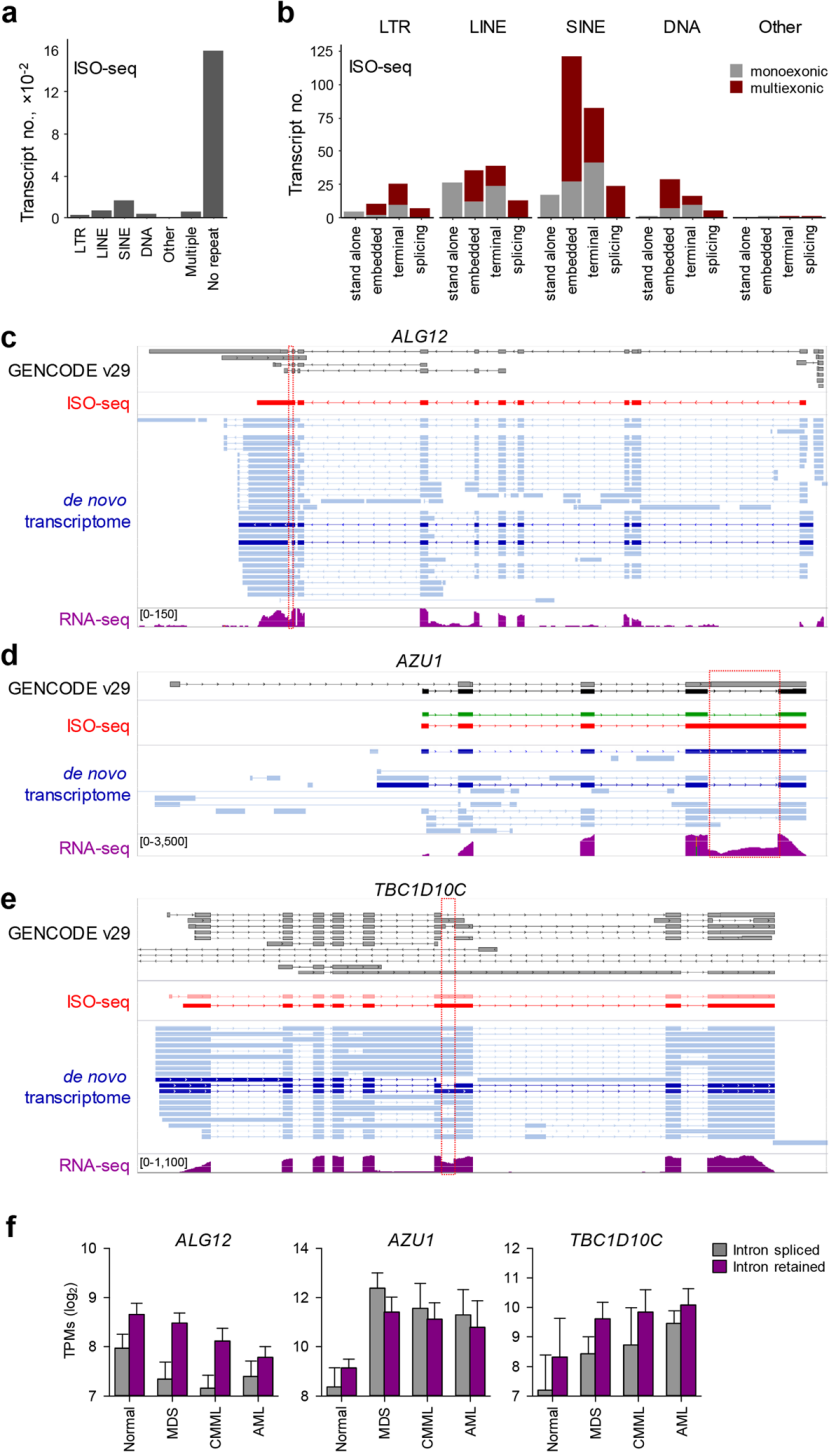
Full-length RNA-seq analysis of dysplastic HSCs. **a** Number of ISO-seq-identified transcripts overlapping with repeats. **b** Representation of multiexonic or monoexonic repeat-overlapping transcripts, according to the transcript structure in ISO-seq transcriptome. **c**–**e** Structure of representative ISO-seq-identified transcripts. Dark red indicates novel, previously unannotated transcripts identified by both ISO-seq and de novo transcript assembly. Previously annotated transcripts are indicated in green in the ISO-seq track and the corresponding GENCODE transcripts in black. De novo-assembled transcripts confirmed by ISO-seq or present in GENCODE v29 annotation are indicated in dark blue. Dotted red boxes indicate the retained introns. RNA-seq read coverage in representative samples is also illustrated as a separate track. **f** Expression of novel intron-retaining ISO-seq-identified and de novo-assembled transcripts (intron retained), in comparison with the respective index fully spliced transcript (intron spliced) in healthy and pre-treatment dysplastic HSCs. HSCs from all untreated patients from both cohorts are shown

Although de novo assembly of the HSC transcriptome did not support a role for EREs in the response to 5-AZA treatment, it did uncover a substantial number of novel, previously unannotated transcripts, which did not necessarily overlap with EREs. In an effort to support the de novo assembly, we additionally performed isoform sequencing (ISO-seq), which has the potential to capture full-length RNAs. Of a total of 1935 full-length RNA transcripts sequenced from dysplastic HSCs, 1269 were previously fully annotated, with the remaining partially annotated or unannotated, and were the predominant transcripts that did not include any ERE (Fig. 4a). ISO-seq-identified transcripts that did overlap with EREs were enriched for embedded or terminal SINEs (Fig. 4b), in agreement with the results of de novo assembly. The intersection of novel ISO-seq and de novo-assembled transcripts identified 49 that were fully supported by both methods, all of which were multiexonic splice variants of gene transcripts.

Novel transcripts included splice variants of *ALG12*, *AZU1* and *TBC1D10C*, all of which were created by intron retention (Fig. 4c–e). *ALG12* encodes a mannosyltransferase with 12 transmembrane domains, and a stop codon in the retained last intron was predicted to cause omission of the last transmembrane domain. Similarly, *AZU1* encodes the secreted peptidase azurocidin and retention of the last intron was predicted to create a C-terminally truncated protein. Lastly, the alternative splice variant of *TBC1D10C* retained introns 6 and 9, rendering it subject to non-sense-mediated decay (NMD). Expression of all 3 genes appeared disease-related, with *ALG12* progressively downregulated from MDS to AML, and *AZU1* and *TBC1D10C* expressed at higher levels in dysplastic than in healthy HSCs (Fig. 4f). Importantly, the intron-retaining variants were expressed at levels equal to or higher than those of the respective reference variants encoding the canonical proteins (Fig. 4f), indicating that intron retention occurred at very high frequency, both in healthy and dysplastic HSCs.

### Expression of alternatively spliced isoforms predictive of 5-AZA therapy outcome

**Fig. 5:**
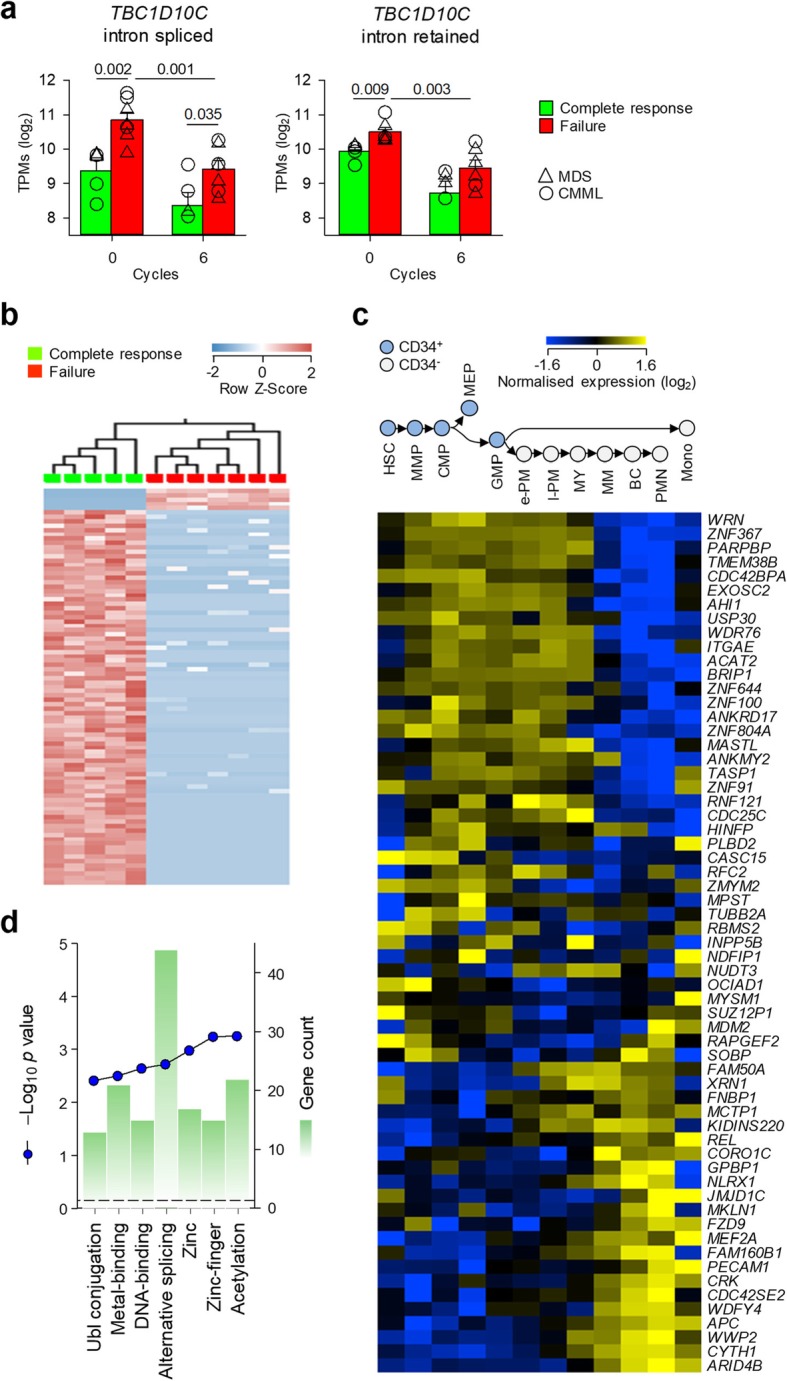
Genes differentiating prospective 5-AZA responses and failures. **a** Expression of index fully spliced transcript (intron spliced) and alternatively spliced (intron retained) *TBC1D10C* transcripts in HSCs isolated prior to 5-AZA treatment in MDS and CMML patients who subsequently responded (complete response) or failed to respond (failure) to 6 cycles of treatment. Only patients from the second cohort are shown, as the first cohort was not sampled after 6 treatment cycles. MDS and CMML patients are indicated by different symbols. **b** Heatmap of the expression of 91 de-novo assembled transcripts in HSCs differentiating prospective treatment responses and failures. Only patients from the second cohort are shown. **c** Expression of genes overlapping treatment outcome-prognostic transcripts at CD34^+^ and CD34^−^ progressive stages of normal haematopoietic development (MPP, multipotential progenitor; CMP, common myeloid progenitor; GMP, granulocyte monocyte progenitor; MEP, megakaryocyte-erythroid progenitor; e-PM, early promyelocyte; l-PM; late promyelocyte; MY, myelocyte; MM, metamyelocyte; BC, band cell; PMN, polymorphonuclear cell; Mono, monocyte). **d** Functional annotation of genes overlapping treatment outcome-prognostic transcripts, according to the Database for Annotation, Visualization and Integrated Discovery (DAVID) v6.8 (https://david.ncifcrf.gov/home.jsp)

Using either the annotated or an extended transcriptome, our analysis indicated that the ERE or gene transcripts that were transcriptionally induced by 5-AZA treatment could not accurately predict its outcome. We therefore asked whether there were any transcripts in the de novo assembly, the expression of which could differentiate clinical responses from failures, irrespective of the modulation of their expression by 5-AZA. Indeed, pre-treatment expression of both intron retention and canonical *TBC1D10C* splice variants was significantly higher in patients who subsequently did not respond to 5-AZA treatment than those who did and appeared downregulated after treatment (Fig. 5a). Direct comparison of pre-treatment samples from patients who then exhibited clinical response or failure identified 91 differentially expressed transcripts (≥ 2-fold, *q* ≤ 0.05), the majority of which (86) were preferentially expressed in prospective responders (Fig. 5a and Additional file 1: Table S6). Of the 86 transcripts that distinguished the pre-treatment state of prospective responders, only one, an LTR element (ERVL-MaLR|MSTB) integrated on chromosome 2, was not overlapping with any annotated genes (Additional file 1: Table S6), reinforcing the stronger correlation of 5-AZA treatment outcome with gene, rather than ERE transcription.

Although equivalent resources for MDS and CMML were not available, analysis of clinical data from AML cohorts of The Cancer Genome Atlas (TCGA) programme revealed that several of genes overlapping with the identified transcripts were prognostic of overall survival, with higher expression of *CASC15*, *CDC25C* and *NLRX1* correlating positively and *RAPGEF2*, *CORO1C*, *NDFIP1*, *DGKA*, *TMEM38B* and *PECAM1* correlating negatively with survival probability (Additional file 2: Figure S7).

Re-analysis of the expression data covering successive stages of normal myeloid development [36] indicated that genes with splice variants overexpressed in prospective responders followed three distinguishable patterns of roughly equal proportion (Fig. 5c). The first included genes progressively increasing expression through the early stages of healthy CD34^+^ HSC differentiation to more specialised progenitors, such as granulocyte monocyte progenitors (GMPs) or megakaryocyte-erythroid progenitors (MEPs) (Fig. 5c). The second included genes that were induced only in later stages of normal myeloid differentiation, starting at myelocytes (MY) and peaking in the most mature monocytes or polymorphonuclear cells (PMN) (Fig. 5c). The third grouping consisted of a minority of genes that demonstrated little change in the expression throughout normal myeloid development (Fig. 5c). By Gene Ontology (GO) analysis, over half of the genes overexpressed in prospective responders were annotated as having at least two splice isoforms and several belonged to zinc finger protein (ZFP) genes (Fig. 5d and Additional file 1: Table S6). These findings suggested that a therapeutic response to 5-AZA treatment correlated with the expression of alternatively spliced variants of developmentally regulated genes, two processes that are connected in normal myeloid differentiation [53].

**Fig. 6:**
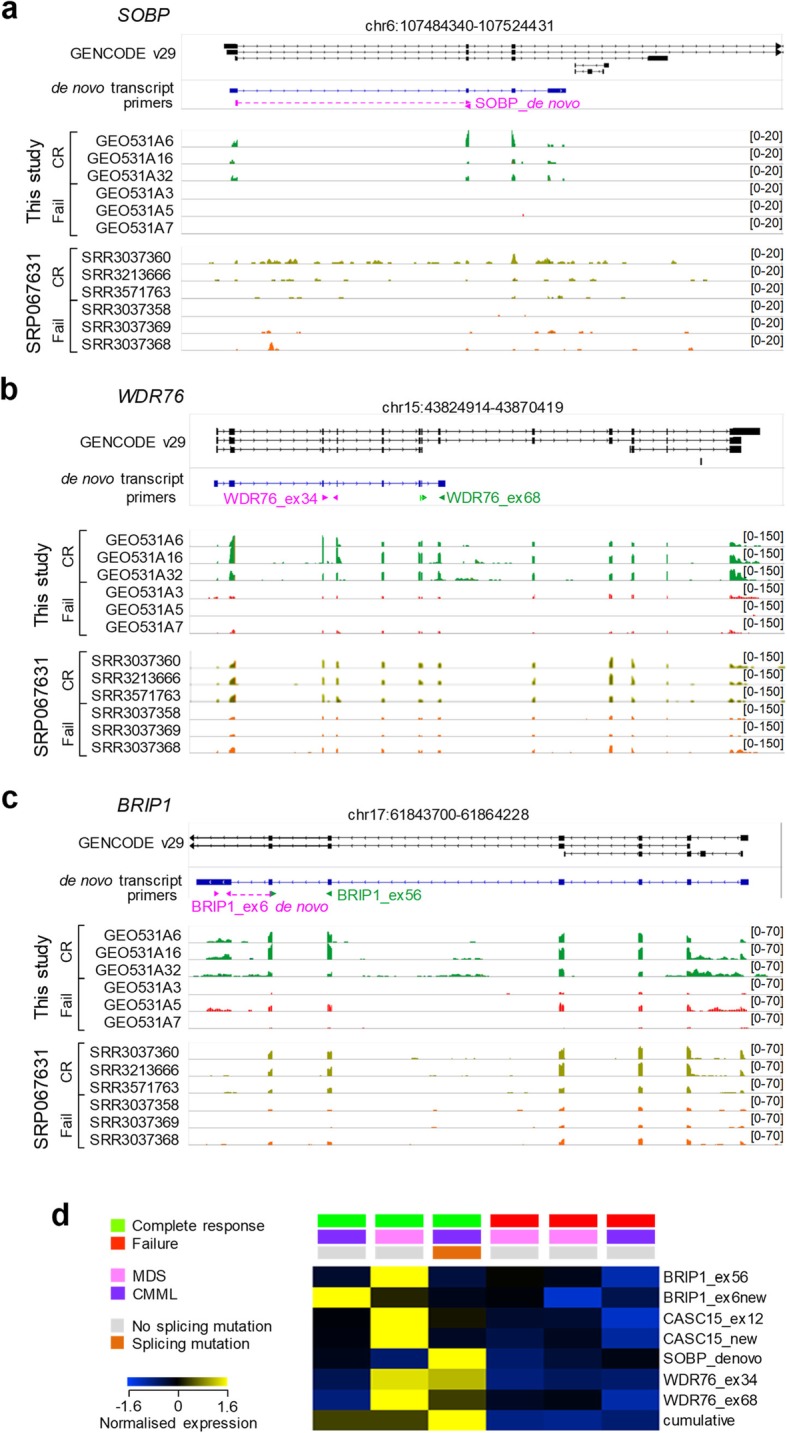
Structure and validation of selected treatment outcome-prognostic transcripts. **a**–**c** Structure of GENCODE-annotated and de novo-assembled *SOBP*, *WDR76* and *BRIP1* transcripts. Location of PCR primers used is represented by pink or green arrowheads for different pairs. RNA-seq read coverage in HSCs isolated prior to 5-AZA treatment from representative samples of MDS (GEO531A16, GEO531A3, GEO531A5) and CMML (GEO531A6, GEO531A32, GEO531A7) patients who subsequently responded (complete response) or failed to respond (failure) to 6 cycles of treatment in our study and in SRP067631. Patient GEO531A32 had a mutation in the spliceosome complex gene *U2AF1*. **d** Expression of qRT-PCR products amplified by primer pairs shown in **a**–**c** and Additional file 2: Figure S8 normalised to *HPRT* expression, in MDS and CMML patients, according to their response to 5-AZA treatment. The cumulative expression is the sum of normalised expression

To further probe the transcriptional features correlating with 5-AZA treatment response or failure, we selected four de novo-assembled transcripts that were overexpressed in our cohort of prospective responders for more detailed analysis. These included a shorter splice variant transcribed from the *CASC15* gene (Additional file 2: Figure S8), encoding several other long non-coding RNA (lncRNA), which were prognostic in AML (Additional file 2: Figure S7). They also included splice variants of *SOBP*, *WDR76* and *BRIP1*, all three of which were truncated versions of the respective protein-coding variants, created by intronic polyadenylation (Fig. 6a–c). Inspection of RNA-seq read coverage agreed with the assembled transcripts structures and expression patterns between clinical responses and failures (Fig. 6a–c and Additional file 2: Figure S8). Elevated expression of splice variants of these genes was not restricted to patients with mutations in splice factors, as the latter represented a minority of the patients in our cohort (Fig. 6a–c). Indeed, mutations in splice factors *U2AF1*, *SF3B1*, *ZRSR2* or *SRSF2* were identified in two MDS patients (in both of which treatment failed) and three CMML patients (with treatment failure, complete response and partial response) (Additional file 1: Table S1) and did not correlate with expression of splice variants or 5-AZA treatment outcome, consistent with prior analyses [54–56].

To extend these observations to the independent SRP067631 cohort, we also plotted on the same scale samples representative of prospective clinical responses and failures in the latter cohort (Fig. 6a–c and Additional file 2: Figure S8). We chose this method of comparison in preference to TPM calculation due to the differences in coverage of these genes in the two datasets and the prevalence of intronic reads in the SRP067631 cohort (example *SOBP*, Fig. 6a). Nevertheless, exon coverage of the selected transcripts in the SRP067631 cohort provided additional support for a correlation of expression with subsequent favourable response to 5-AZA (Fig. 6a–c and Additional file 2: Figure S8). Lastly, transcript structures and expression patterns were further confirmed by qRT-PCR in samples from patients with subsequent clinical response or failure, using primer pairs specific for shared or novel exons (Fig. 6d). Samples from prospective responders expressed high levels of one or more of the selected splice variants, but not all at the same time (Fig. 6d). In contrast, samples from prospective failures were consistently negative (Fig. 6d), and the normalised sum of their expression could distinguish the two groups (*p* = 0.0164, two-tailed *t* test).

## Discussion

Despite its potential clinical utility, our understanding of the precise mode of epidrug action and, therefore, our ability to predict treatment responses and failures in MDS and related cancers is still limited. Here, we have examined the possible involvement of ERE derepression in the therapeutic response to 5-AZA treatment, recently suggested by a number of largely in vitro studies [24, 25, 28, 29]. Our findings argue against ERE modulation and subsequent activation of innate immunity through ‘viral mimicry’ in HSCs as determinants of the in vivo response of MDS and CMML patients to 5-AZA treatment. Instead, the in-depth analysis of healthy and dysplastic HSCs offered by de novo transcript assembly reveals extensive splicing diversity of developmentally regulated genes with superior prognostic properties of the response to 5-AZA.

The use of hidden Markov models (HMMs) to represent ERE families can improve the accuracy and sensitivity of ERE annotation [57]. RNA-seq read counting based on such methods has successfully captured modulation of ERE representation in the total transcriptome in healthy and transformed murine and human primary B lymphocytes [45], as well as in human cancer cell lines and primary MDS HSCs treated with 5-AZA in vitro [24–28] and in bone marrow MDS and CMML HSCs following in vivo treatment with 5-AZA in this study. Although these methods give an accurate estimate of the overall inclusion of EREs in RNA transcripts, it is important to note that they cannot supply information regarding the nature or structure of the individual ERE-incorporating transcripts. This is because the majority of EREs in the genome are not distinct transcriptional units. Instead, most ERE-mapping reads are likely to belong to longer RNA transcripts of protein-coding and non-coding genes. For example, Alu-containing transcripts can derive from stand-alone Alu elements, transcribed by RNA polymerase III, or from Alu elements embedded in larger transcripts, typically in the 3′UTR, transcribed by RNA polymerase II [58]. Regulation of the two types of Alu-overlapping transcripts would be through different mechanisms, but standard read counting workflows cannot readily distinguish between the two, and it is often the case that the apparent increases in ERE-mapping read representation are simply due to the upregulation of genic transcripts, in which EREs are embedded, rather than their independent upregulation.

Absolute quantitation of ERE-overlapping transcripts thus requires knowledge of transcript structure. Indeed, recent studies have indicated a high degree of transcriptional diversity in health and disease, which is not yet fully captured in existing transcriptome annotations [52, 59–64]. The de novo transcript assembly method we employed in this study, when applied to 32 other types of cancer, uncovered thousands of previously unannotated or partially annotated transcripts overlapping with LTR elements and expressed specifically in cancer [52], underscoring the potential of the approach. Consistent with prior efforts, this comprehensive view of ERE modulation in HSCs from MDS and related cancers did identify transcripts transcriptionally responsive to 5-AZA treatment in vivo. Compared with most other cell types or transformation phenotypes, where our method has captured increased ERE activity [52], 2 key aspects distinguish dysplastic HSCs. Firstly, compared with differentiated haematopoietic cells, such as T cells, both healthy and dysplastic HSCs express higher levels of specific ERVs that were previously found to be responsive to 5-AZA treatment, such as *ERV3-1*. This elevated level of ERV transcription in HSCs in general is present in untreated MDS and CMML HSCs and could blunt modulation by 5-AZA. Secondly, compared with healthy HSCs, we found that dysplastic HSCs display reduced overall transcriptional activity, consistent with independent reports [46]. Therefore, the lack of overt ERE activation by 5-AZA treatment of MDS and CMML HSCs, above levels seen in healthy HSCs, is not likely due to the lack of sensitivity in our detection and quantitation methods, but rather due to the unique features of HSCs in general and the global transcriptional repression that characterises dysplastic HSCs in particular. These two properties of HSCs may also underlie the lack of correlation between ERE induction in this cell type with the outcome of 5-AZA therapy, which seems to be at odds with observations in other cancers or cell types [24, 25]. Despite the atypical ERE and gene suppression in dysplastic HSCs, in vivo treatment with 5-AZA did induce ERE transcription, as would be expected for an epidrug. However, restoration of ERE transcription in MDS and CMML HSCs was partial, not fully reaching transcription in healthy HSCs. More pertinently, none of the previously annotated or novel 5-AZA treatment-responsive ERE-overlapping transcripts was reliably further upregulated specifically in treatment responses than in failures, and similar findings were obtained for ERE families, including those previously suggested in the literature [24, 25, 28, 47–49].

Concordant with the absence of an ERE transcriptional signature specific to a favourable outcome of 5-AZA therapy, our analysis did not point to an IFN signature specific to this outcome. Moreover, whilst ERE induction by 5-AZA treatment was detectable, but not restricted to responding patients, induction of an IFN response was not detectable in the transcription of typical ISGs, IFN-inducible LTR elements or ISGs previously shown to be induced in cell lines by 5-AZA in vitro [50]. It is conceivable that 5-AZA treatment of MDS or CMML patients does not trigger an intrinsic IFN response in HSCs. However, as our first samples were obtained as late as 6 days after the end of the first round of 5-AZA treatment, we cannot exclude the possibility that a transient IFN response was induced at earlier time points.

The lack of a sustained IFN signature in 5-AZA-treated MDS and CMML patients here agrees with the previous independent analysis of the SRP067631 cohort, which also lacked an IFN signature [16]. Seemingly at odds, however, is the absence in our cohort of 5-AZA responders of inflammation-related genes that Unnikrishnan et al. previously found induced by 5-AZA in specifically in the responders of the SRP067631 cohort [16]. It should be noted that these genes are not only expressed in inflammation or following 5-AZA treatment. Indeed, the intersection of the 302 genes induced by 5-AZA in CD34^+^ cells in vivo [16] and the 401 genes induced by 5-AZA in breast, colorectal or ovarian cell lines [50] was minimal (19 out of 302 genes) and included genes, such as *IL1R1*, *CTSS*, *PLA2G7*, *PTAFR*, *CD1D*, *CD36* and *TLR3*, expressed during inflammation, as well as highly dynamically during normal myeloid cell differentiation. It is, therefore, possible that the apparent induction of these genes specifically in the responders of the SRP067631 cohort simply reflects restored myelopoiesis. Consistent with this notion, the HSC isolation method used here, but not in prior studies, specifically excludes CD45^+^CD34^+^ cells, a sizeable fraction of more differentiated CD45^+^ cells that also express CD34. Unless excluded, these differentiated cells would contribute to gene expression profiles, particularly of developmentally regulated genes. Thus, the choice of cell type might be an important determinant of the observed effect of 5-AZA treatment. Poor correlation between an IFN response and the outcome of 5-AZA therapy is also suggested by a recent study reporting higher expression of the necroptosis mediator *MLKL* in untreated MDS and CMML HSCs than in healthy HSCs [65]. High *MLKL* expression positively correlated with a cytokine release and a proinflammatory response in MDS and CMML HSCs and was reduced, rather than increased following 5-AZA treatment [65].

A lack of IFN response in these studies might be specific to HSCs. Indeed, a notable difference between HSCs and differentiated haematopoietic cells is the constitutive expression of ISGs and sensitivity to IFN stimulation. Similarly to several other types of stem cell, which constitutively express a range of ISGs [51], heathy and untreated dysplastic HSCs exhibited clearly elevated constitutive transcription of ISGs previously reported responsive to 5-AZA treatment, when compared with T cells. Moreover, diverse types of stem cell, including embryonic, neural, pancreatic, mesenchymal and haematopoietic have been described to lack responsiveness to IFN stimulation [51]. In contrast, differentiated cells lose the constitutive expression of ISGs that characterises HSCs and become responsive to IFN stimulation [51]. These unique features of HSCs could, therefore, account for the apparent lack of IFN response in this cell type following 5-AZA treatment in vivo. However, our study has certain limitations. Our focus on highly purified bone marrow HSCs was necessary to interrogate the possible effect of 5-AZA on the affected cell type in MDS and CMML, but does not allow extrapolation to other, more differentiated cell types, in which the IFN pathway is functional. Furthermore, the use of purified bone marrow HSCs restricted the numbers of patient samples. The relatively low numbers of individual patient data points generated here, or are publicly available may thus lack the statistical power to capture smaller effects of 5-AZA on inflammatory gene transcription in HSCs.

Whilst our de novo assembly and full-length RNA-seq did not support a role for annotated or novel ERE transcripts in the therapeutic response to 5-AZA, it did highlight the extensive presence of splice isoforms of protein-coding and non-coding genes, particularly ones created by intron retention. Intron-retaining isoforms were a substantial fraction of certain protein-coding gene transcripts and could impact protein function, either due to premature stop codons in the retained introns, leading to the production of truncated proteins (e.g. *ALG12* and *AZU1*) or to NMD of aberrantly spliced mRNA (e.g. *TBC1D10C*). Loss-of-function *ALG12* mutations cause *ALG12*-congenital disorder of glycosylation [66], but this gene has not been previously associated with cancer. *AZU1* expression, which is upregulated in MDS, CMML and AML, has been linked with certain other myeloproliferative neoplasms [42], as well as renal cell and prostate cancer [67, 68]. *TBC1D10C*, which is also upregulated in MDS, CMML and AML, has recently been suggested to correlate with responsiveness to cancer immunotherapy [69]. *CASC15* (*cancer susceptibility 15*; previously annotated as *LINC00340*) is a lncRNA with reported tumour suppressor properties in melanoma, neuroblastoma and acute leukaemia [70–72], which is also associated with higher survival probability in AML. WDR76 was also recently reported as a tumour suppressor in hepatocellular carcinoma [73]. Lastly, BRIP1, which interacts with and is necessary for the function of BRCA1, is a tumour suppressor, and loss-of-function germline mutations increase the risk of breast and ovarian cancers [74, 75].

Given the association of these genes with other cancers, perturbations of their mRNA or protein levels could account for or contribute to the association with prognosis in AML or outcome of 5-AZA therapy in MDS and CMML, and this warrants further investigation. However, a simpler explanation for the observed association would be that overall expression and alternative splicing of these genes reflects the developmental progression that characterises normal myelopoiesis. A number of observations support this notion. Firstly, the genes that differentiate healthy HSCs from untreated dysplastic HSCs belong to divergent haematopoietic lineages (lymphoid and myeloid, respectively), consistent with well-established differentiation defects in myelodysplasias. Secondly, the majority of the genes that distinguish prospective 5-AZA responses and failures are developmentally regulated and, hence, are expressed in distinct waves through myelopoiesis. Thirdly, extensive intron retention is also a developmentally regulated process during normal myelopoiesis [53], as well as erythropoiesis [76], and is thought to reduce mRNA and protein production.

These observations support a model whereby the outcome of 5-AZA therapy is determined by the degree of residual or ongoing haematopoietic development, reflected in the expression of developmentally regulated genes and the extent of alternative splicing. This model is supported by findings that lower-risk and higher-risk MDS are characterised by expansion of HSCs at different stages of myeloid development [77]. It also fits with independent observations linking increased HSC quiescence with resistance to decitabine or 5-AZA therapy [14, 16]. Expression of *ITGA5*, thought to be required for the maintenance of quiescence, was not as strongly associated with therapy failure in this study, as previously reported [16]. Higher expression of *ITGA5* has recently been correlated with higher expression of *RIPK1*, an adverse prognostic factor in untreated MDS and CMML patients [65], and it is, therefore, possible that it associates with more aggressive disease independently of treatment. The balance between quiescent and active HSCs reflects ongoing haematopoiesis [78], and, as the incorporation of these nucleoside analogues requires DNA replication, HSC quiescence reduces epidrug efficacy. Supporting a role for the degree of nucleoside analogue incorporation, the expression of cytidine deaminase, which reduces the bioavailability of 5-AZA, and of other enzymes involved in 5-AZA metabolism, has also been linked with resistance to 5-AZA therapy [19, 79].

The extended healthy and dysplastic HSC transcriptome we provide in this study establishes the link between ongoing HSC differentiation and the response to 5-AZA therapy, independently of EREs, and will form the basis for biomarker analysis in larger cohorts, as they become available.

## Conclusions

Using three separate methods of transcriptome analysis, our approach found no evidence to support the prevalent hypothesis that induction of ERE transcription is linked to the success of epidrug therapy in MDS or CMML. Instead, the comprehensive assembly of transcripts expressed by healthy and dysplastic HSCs uncovered the pervasiveness of alternative splicing, particularly intron retention, of protein-coding and non-coding gene transcripts. This improved view of HSC transcriptional diversity, in turn, revealed the transcriptional signatures that predict the response of MDS and CMML patients to 5-AZA treatment. The overall picture that emerges is that the outcome of 5-AZA treatment is determined by the degree of residual or ongoing HSC differentiation, reflected in the pre-treatment expression and alternative splicing of developmentally regulated gene transcripts, many of which are novel candidates for further analysis.

## Supplementary information

**Additional file 1:** **Table S1**. Details of patients recruited in this study. **Table S2**. Details of patient samples used in this study. **Table S3**. PCR primers used in this study. **Table S4**. 479 elements differentially expressed between healthy and dysplastic HSCs. **Table S5**. Interferon-stimulated genes (ISG) and LTR elements (IS-LTR) used in this study. **Table S6**. 91 transcripts differentially expressed between responders and failures prior to 5-AZA treatment.

**Additional file 2:** **Figure S1**. Gating strategy for cell sorting. **Figure S2**. Expression changes of selected individual EREs in CD34^+^ HSCs upon 5-AZA treatment. **Figure S3**. Expression changes of selected ERE families in CD34^+^ HSCs upon 5-AZA treatment. **Figure S4**. Lack of interferon signature in MDS and CMML HSCs cells in response to 5-AZA treatment. **Figure S5**. Expression of inflammation-related genes MDS and CMML HSCs cells in response to 5-AZA treatment. **Figure S6**. Elevated expression of ISGs and ERVs in healthy and untreated dysplastic HSCs. **Figure S7**. Survival probability in AML according to expression of the indicated prognostic transcripts. **Figure S8**. Structure of the treatment outcome-prognostic transcript *CASC15*.

## Data Availability

Datasets generated and analysed during the current study are available at the EMBL-EBI repository (www.ebi.ac.uk/arrayexpress) under accession numbers E-MTAB-8208 (RNA-seq) and E-MTAB-8195 (ISO-seq). Previously deposited datasets analysed during the current study include RNA-seq data from human CD34^+^ HSCs [16], available at SRA (www.ncbi.nlm.nih.gov/sra) under accession number SRP067631, and microarray data from normal human haematopoiesis [36], obtained from the BloodSpot data portal (www.bloodspot.eu), with the original data available at the GEO repository (www.ncbi.nlm.nih.gov/geo) under accession number GSE42519.
