# Supplementary material for: Epigenetic therapy of myelodysplastic syndromes connects to cellular differentiation independently of endogenous retroelement derepression
Source: Genome Med. 2019 Dec 23;11:86. doi: 10.1186/s13073-019-0707-x (PMC6929315; doi:10.1186/s13073-019-0707-x)
Supplement: Supplementary file 2 — Additional file 2: Figure S1. Gating strategy for cell sorting. Figure S2. Expression changes of selected individual EREs in CD34+ HSCs upon 5-AZA treatment. Figure S3. Expression changes of selected ERE families in CD34+ HSCs upon 5-AZA treatment. Figure S4. Lack of interferon signature in MDS and CMML HSCs cells in response to 5-AZA treatment. Figure S5. Expression of inflammation-related genes MDS and CMML HSCs cells in response to 5-AZA treatment. Figure S6. Elevated expression of ISGs and ERVs in healthy and untreated dysplastic HSCs. Figure S7. Survival probability in AML according to expression of the indicated prognostic transcripts. Figure S8. Structure of the treatment outcome-prognostic transcript CASC15. [file 13073_2019_707_MOESM2_ESM.pdf]

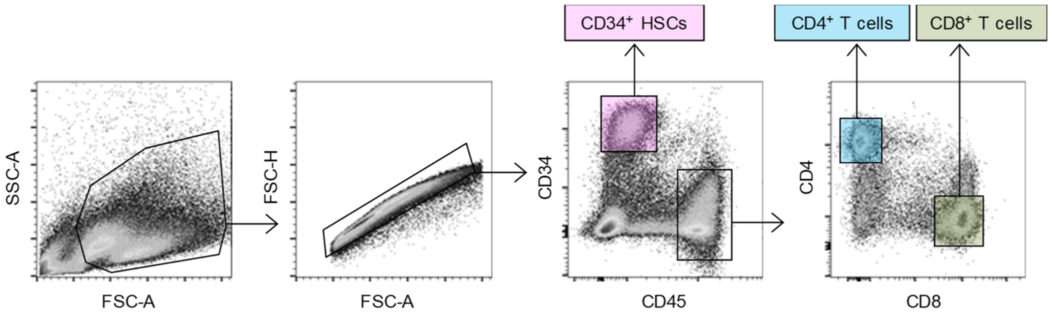

Figure S1. Gating strategy for cell sorting. Flow cytometric identification of bone marrow CD34$^+$ CD45$^-$ HSCs, CD4$^+$ T cells and CD8$^+$ T cells isolated by cell sorting from bone marrow aspirates.

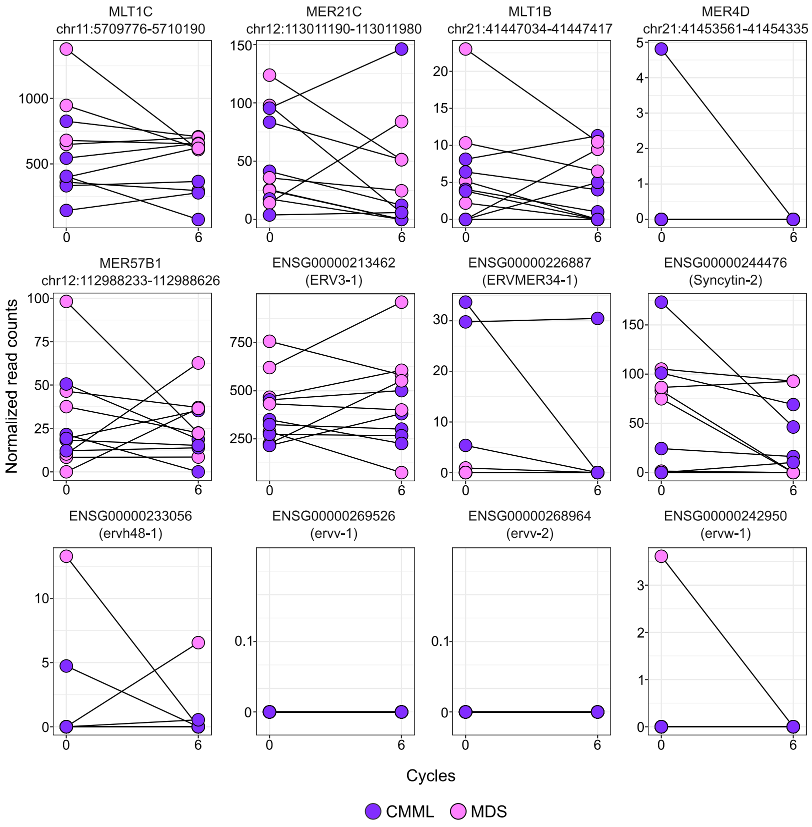

	ALL 0vs6	CR 0vs6	Fail 0vs6
MLT1C chr11:5709776-5710190	ns	ns	ns
MER21C chr12:113011190-113011980	ns	ns	ns
MLT1B chr21:41447034-41447417	ns	ns	ns
MER4D chr21:41453561-41454335	ns	ns	ns
MER57B1 chr12:112988233-112988626	ns	ns	ns
ENSG00000213462 (ERV3-1)	ns	ns	ns
ENSG00000226887 (ERVMER34-1)	ns	ns	ns
ENSG00000244476 (Syncytin-2)	ns	ns	0.0458
ENSG00000233056 (ervh48-1)	ns	ns	ns
ENSG00000269526 (ervv-1)	ns	ns	ns
ENSG00000268964 (ervv-2)	ns	ns	ns
ENSG00000242950 (ervw-1)	ns	ns	ns

Figure S2. Expression changes of selected individual EREs in $CD34^+$ HSCs upon 5-AZA treatment. These individual retroelements have been previously suggested in the literature to be activated by 5-AZA or other epidrugs. The table shows statistical comparisons between expression levels before and after 6 cycles of 5-AZA treatment (paired t-test). Statistical significance has been analysed for responders (CR) and non-responders (Fail) separately and together (ALL) (ns, not significant). Only patients from the second cohort are shown, as the first cohort was not sampled after 6 treatment cycles, and lines connect the values for a given patient.

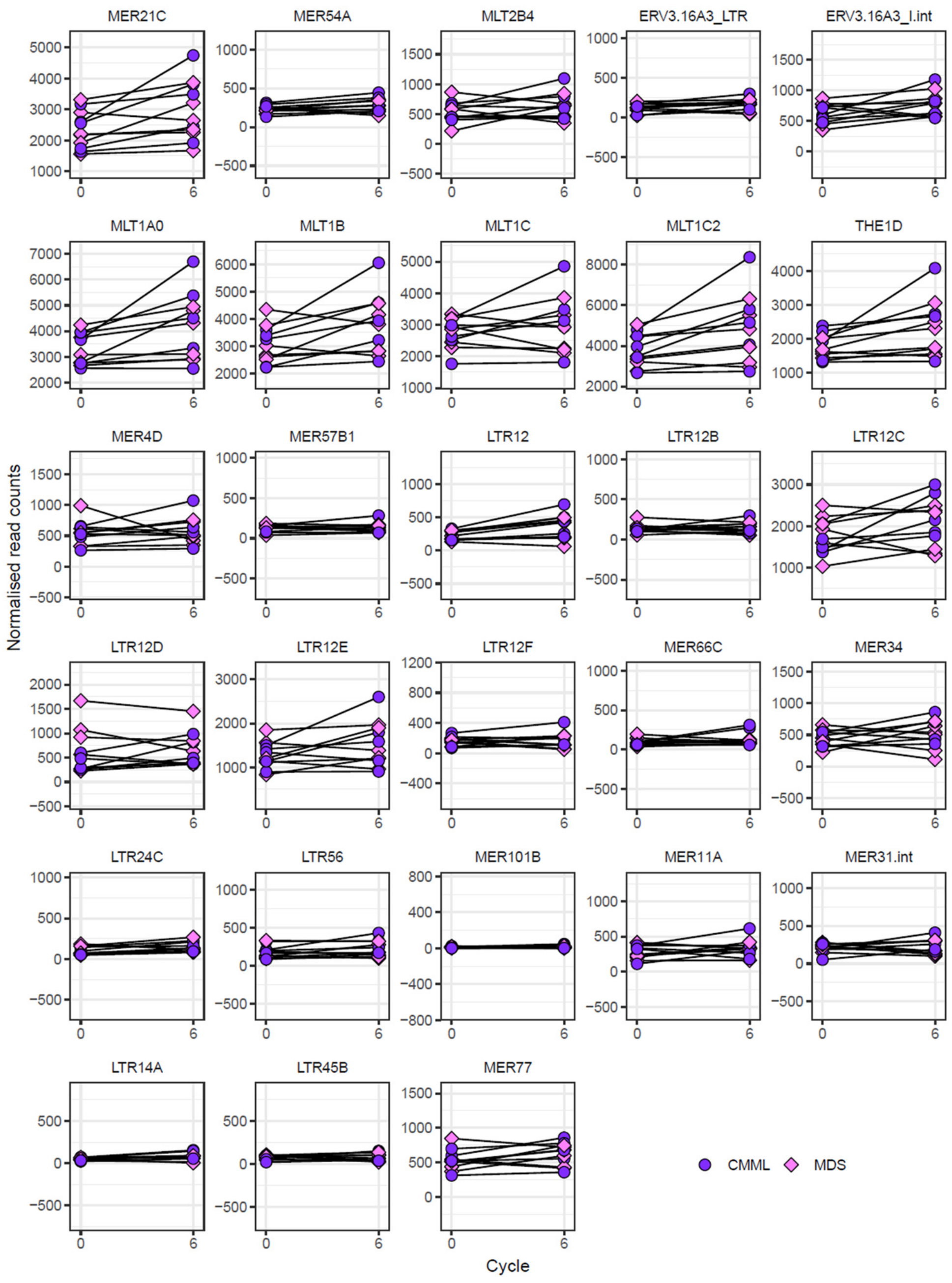

	MER21C	MER54A	MLT2B4	ERV3-16A3_LTR	ERV3-16A3_I-int	MLT1A0	MLT1B	MLT1C	MLT1C2	THE1D
ALL 0vs6	0.0173	ns	ns	0.0342	ns	0.0145	0.0297	ns	0.0118	0.0245
CR 0vs6	ns	0.0073	ns	ns	ns	ns	0.0304	ns	ns	ns
Fail 0vs6	ns	ns	ns	ns	ns	ns	ns	ns	ns	ns

	MER4D	MER57B1	LTR12	LTR12B	LTR12C	LTR12D	LTR12E	LTR12F	MER66C	MER34
ALL 0vs6	ns	ns	0.0051	ns	ns	ns	ns	ns	ns	ns
CR 0vs6	ns	ns	0.0339	ns	ns	ns	ns	ns	ns	ns
Fail 0vs6	ns	ns	ns	ns	ns	ns	ns	ns	ns	ns

	LTR24C	LTR56	MER101B	MER11A	MER31.int	LTR14A	LTR45B	MER77
ALL 0vs6	0.0124	ns	ns	ns	ns	ns	ns	ns
CR 0vs6	0.0338	ns	ns	ns	ns	ns	ns	ns
Fail 0vs6	ns	ns	ns	ns	ns	ns	ns	ns

Figure S3. Expression changes of selected ERE families in $CD34^+$ HSCs upon 5-AZA treatment. These families comprise the individual retroelements from Fig. S2. The table shows statistical comparisons between expression levels before and after 6 cycles of 5-AZA treatment (paired t-test). Statistical significance has been analysed for responders (CR) and non-responders (Fail) separately and together (ALL) (ns, not significant). Only patients from the second cohort are shown, as the first cohort was not sampled after 6 treatment cycles, and lines connect the values for a given patient.

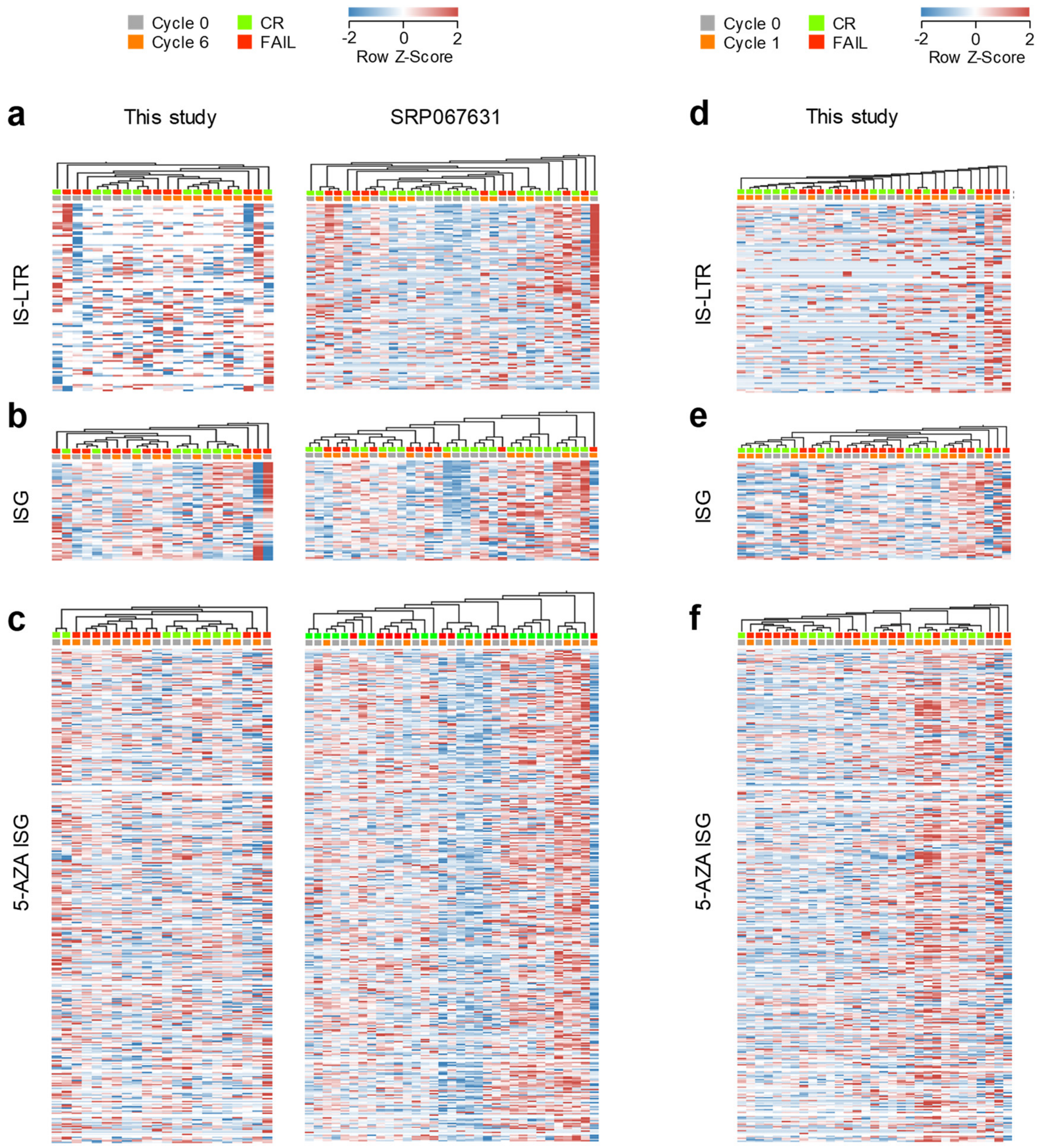

Figure S4. Lack of interferon signature in MDS and CMML HSCs cells in response to 5-AZA treatment. **(a-c)** Interferon inducible gene expression in MDS and CMML HSCs cells before and after 6 cycles of 5-AZA treatment. Only patients from the second cohort are shown, as the first cohort was not sampled after 6 treatment cycles. **(d-f)** Interferon inducible gene expression in MDS and CMML HSCs cells before and after 1 cycle of 5-AZA treatment. Patients from both cohorts are shown. **(a,d)** Hierarchically clustered heatmap of expression of interferon-responsive LTR retroelements (IS-LTR) (Table S4; previously defined in Attig et al. 2017) in our study and in SRP067631. **(b,e)** Hierarchically clustered heatmap of expression of a complied list of 58 interferon inducible genes (ISG) (Table S4) in our study and in SRP067631. **(c,f)** Hierarchically clustered heatmap of expression of 5-AZA inducible immune-relate and cancer-testis antigen genes (5-AZA ISG) (previously defined in Li et al. 2014) in our study and in SRP067631.

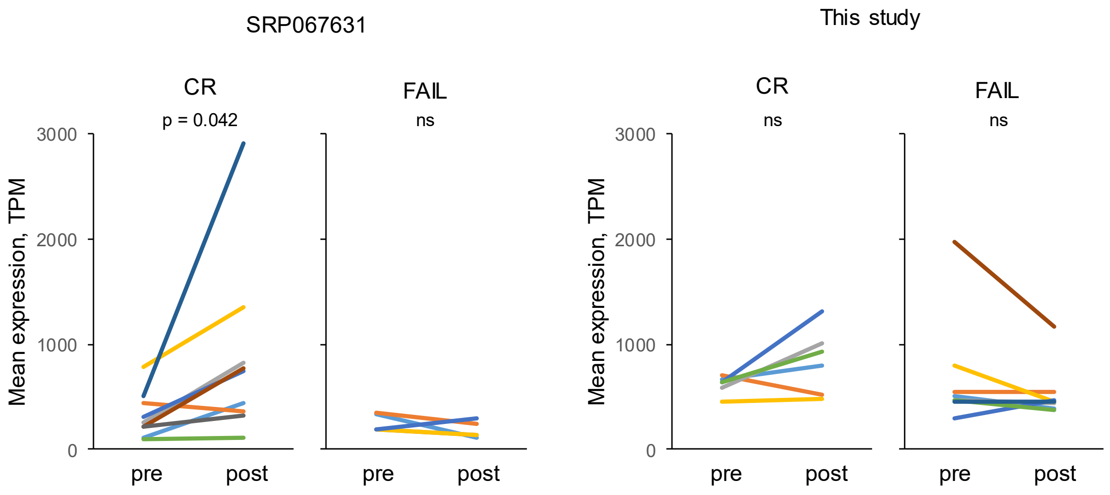

Figure S5. Expression of inflammation-related genes MDS and CMML HSCs cells in response to 5-AZA treatment. The 302 genes that were previously identified by Unnikrishnan et al. 2017 to be induced after 6 cycles of 5-AZA treatment preferentially in responders, were interrogated in data from the original study SRP067631 and from our study. Plots show the averaged mean expression of these 302 genes separately in responders (CR) and non-responders (FAIL) in the two studies. Different coloured lines represent individual patients. Only patients from the second cohort are shown for our study, as the first cohort was not sampled after 6 treatment cycles. Statistical comparisons were made using Mann-Whitney Rank Sum Tests.

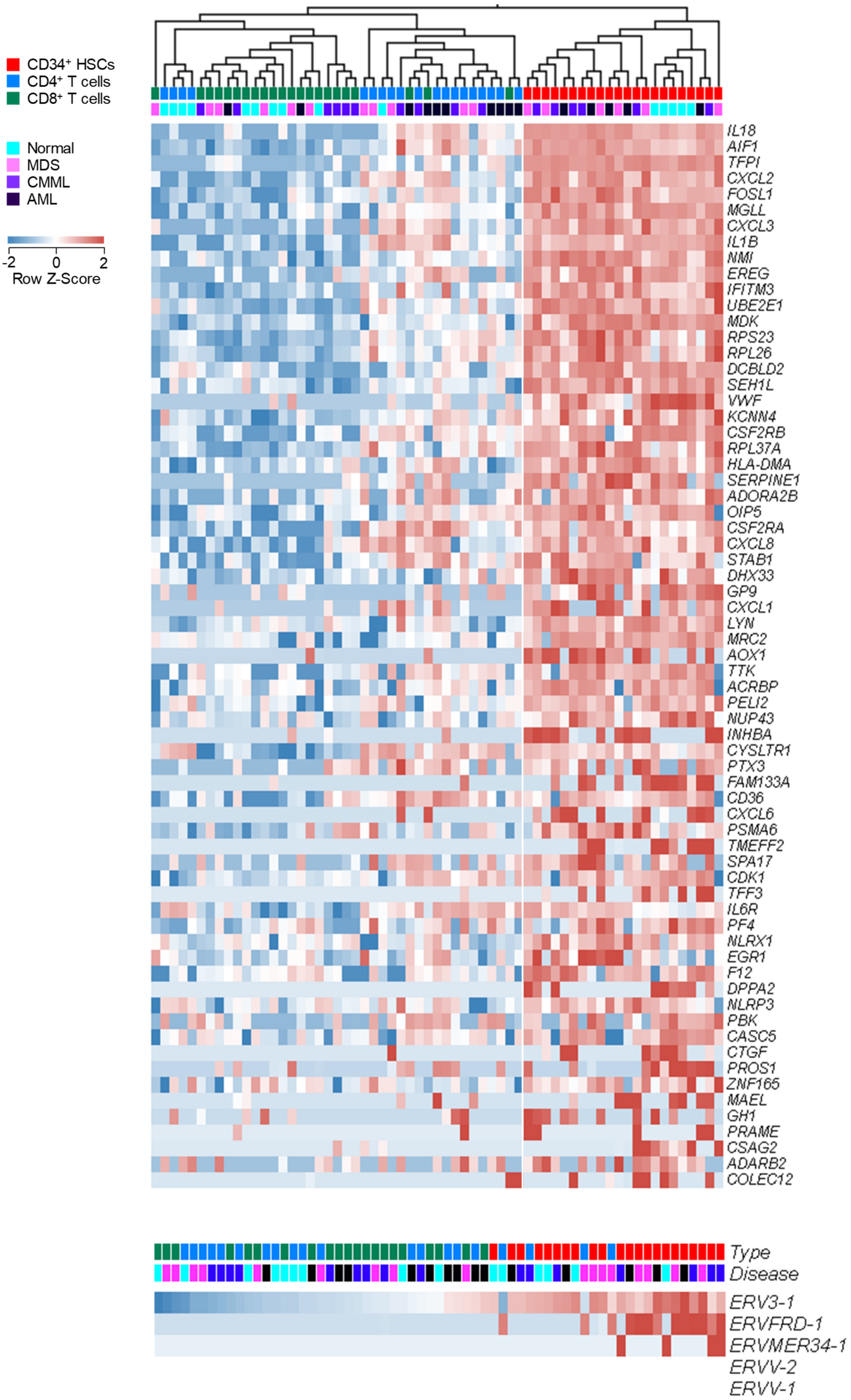

Figure S6. Elevated expression of ISGs and ERVs in healthy and untreated dysplastic HSCs. Hierarchically clustered heatmap of expression of 5-AZA inducible immune-relate and cancer-testis antigen genes (5-AZA ISG) (previously defined in Li et al. 2014) (*top*) and of select ERVs (*bottom*) in CD34+ HSCs and the indicated type of T cell from healthy donors or untreated MDS, CMML and AML patients in our study.

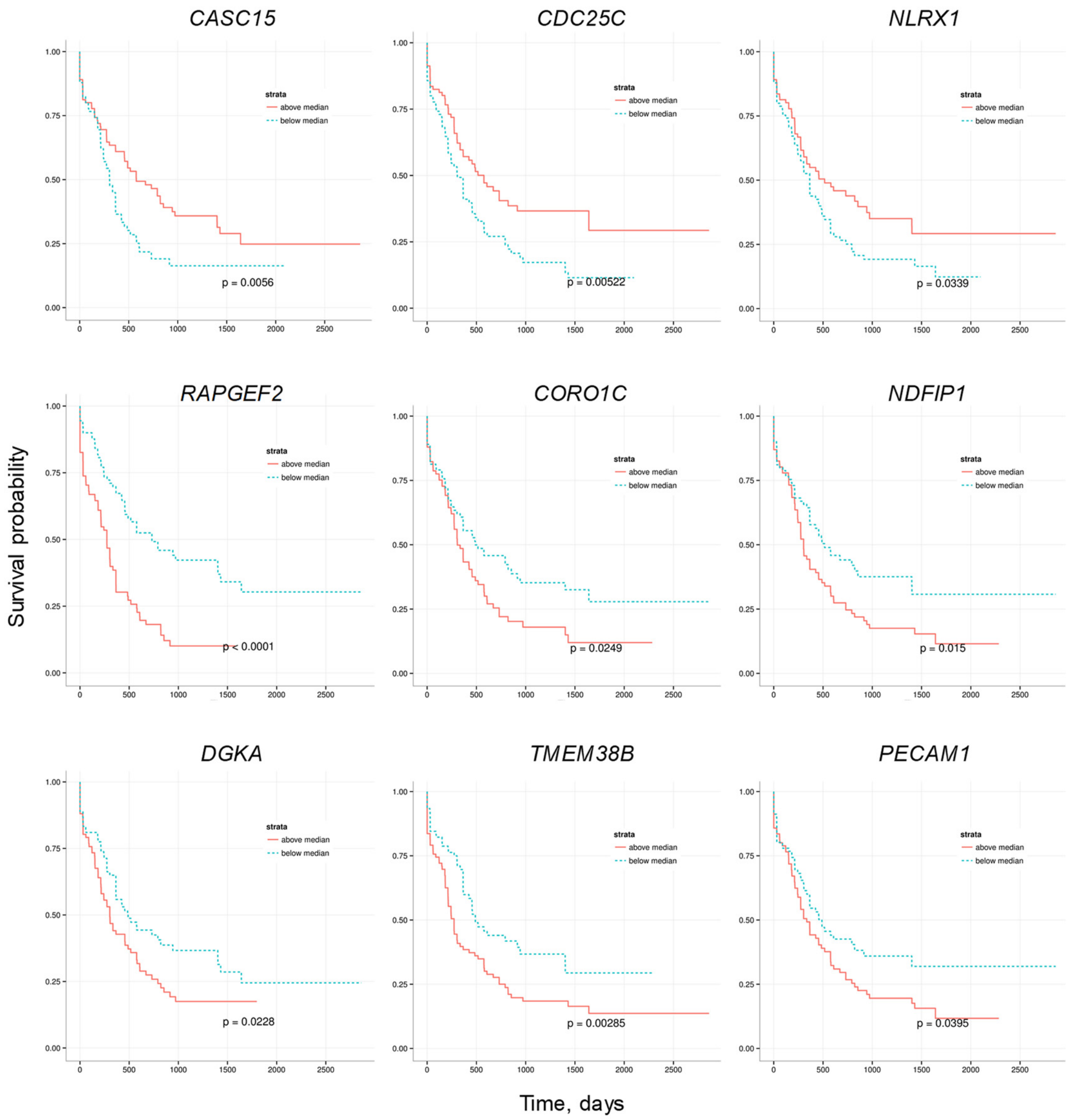

Figure S7. Survival probability in AML according to expression of the indicated prognostic transcripts. Kaplan-Meier survival plots for index genes transcripts overlapping *de novo* assembled treatment outcome-prognostic transcripts in a TCGA cohort (172 patients) stratified by expression above (red) and below (cyan) the median of each transcript.

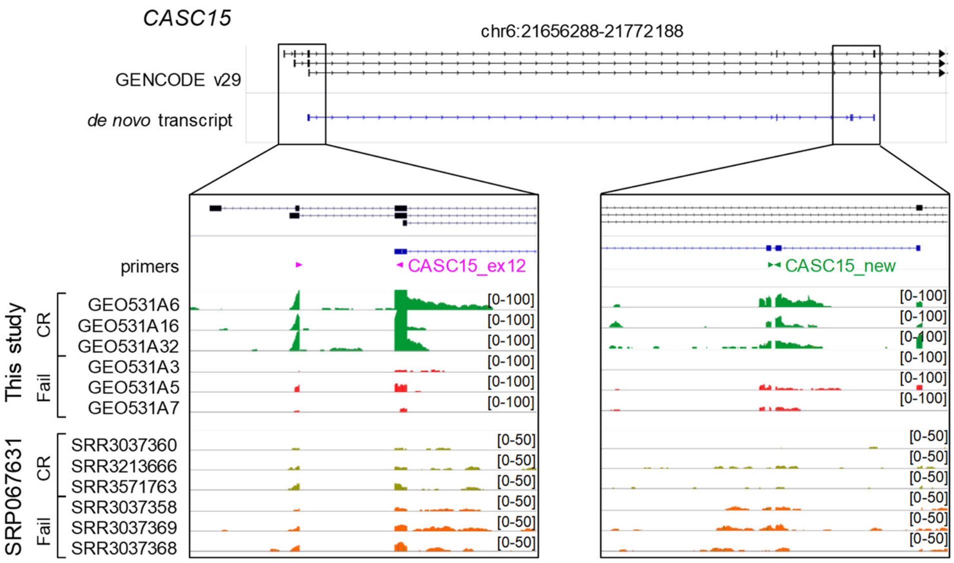

Figure S8. Structure of the treatment outcome-prognostic transcript *CASC15*. Structure of GENCODE annotated and *de novo* assembled *CASC15* transcripts. Only part of the 5' end of GENCODE annotated *CASC15* transcripts are shown. Location of PCR primers used is represented by pink or green arrows for different pairs. RNA-seq read coverage in HSCs isolated prior to 5-AZA treatment from representative samples of MDS (GEO531A16, GEO531A3, GEO531A5) and CMML (GEO531A6, GEO531A32, GEO531A7) patients who subsequently responded (complete response) or failed to respond (failure) after 6 cycles of treatment in our study and in SRP067631. Patient GEO531A32 had a mutation in the spliceosome complex gene *U2AF1*.
